# Governmental Incentives, Satisfaction with Health Promotional Materials, and COVID-19 Vaccination Uptake among Community-Dwelling Older Adults in Hong Kong: A Random Telephone Survey

**DOI:** 10.3390/vaccines10050732

**Published:** 2022-05-06

**Authors:** Zixin Wang, Yuan Fang, Fuk-yuen Yu, Paul Shing-fong Chan, Siyu Chen

**Affiliations:** 1 JC School of Public Health and Primary Care, The Chinese University of Hong Kong, Hong Kong 999077, China; benfyyu@cuhk.edu.hk (F.-y.Y.); pchan@link.cuhk.edu.hk (P.S.-f.C.); chensiyu@link.cuhk.edu.hk (S.C.); 2 Department of Health and Physical Education, The Education University of Hong Kong, Hong Kong 999077, China; lunajoef@gmail.com

**Keywords:** COVID-19 vaccination uptake, older adults, incentives, health promotional materials, perceptions, decisional conflicts, China

## Abstract

COVID-19 vaccination is proven to be effective and safe for older adults. This study investigated the impacts of incentives and health promotional materials provided by the government on the completion of the primary COVID-19 vaccination series among older adults in Hong Kong. Participants were Chinese-speaking community-dwelling adults aged ≥65 years. Telephone numbers were randomly selected from up-to-date Hong Kong telephone directories. A total of 440 participants completed the telephone interview. Logistic regression models were fitted. Among the participants, 58.4% had completed the primary COVID-19 vaccination series. Most participants believed that incentives provided by the government had almost no impact on increasing their motivation to receive COVID-19 vaccination, and less than half thought that vaccination promotional materials produced by the government could address their concerns and help them make decisions. After adjustment for significant background characteristics, we found perceived higher impacts of the incentives and belief that vaccination promotional materials produced by the government could address their concern and were helpful for them to make decisions to be associated with a higher rate of completion of primary COVID-19 vaccination series. Perceptions supporting COVID-19 vaccination and less decisional conflict regarding the choice of vaccine were also positively associated with the dependent variable. Government should develop incentives and health promotional materials tailored to the needs of older adults.

## 1. Introduction

Vaccination and other behavioral preventive measures can help eradicate the ongoing coronavirus disease 2019 (COVID-19) pandemic [1]. International health authorities advocate for all people without a contraindication to receive COVID-19 vaccination [2]. In Hong Kong, where the study was conducted, the government has been implementing a free territory-wide vaccination program for all people aged 3 years or above [3]. One inactivated vaccine (CoronaVac, Sinovac) and one mRNA vaccine (Comirnaty, Fosun-BioNTech, equivalent to Pfizer-BioNTech outside China) are available in Hong Kong [3]. In November 2021, when this study began, 4.7 million people in Hong Kong had received at least one dose of the COVID-19 vaccine, accounting for 69.9% of the population aged 12 years or above [4].

Globally, increasing age is a leading risk factor of COVID-19 mortality [5]. Local data showed that persons aged ≥60 years represented up to 95% of COVID-19 mortality, with a high crude case fatality ratio (7.4–26.0%) [6]. COVID-19 vaccination is proven to be effective and safe in preventing deaths and other severe consequences caused by COVID-19 among older adults [7]. Across countries, older adults have been listed as the top priority group since the rollout of the vaccination program [3]. Worldwide, older adults had a high level of acceptance of COVID-19 vaccination. The prevalence of willingness to receive COVID-19 vaccination among older adults was 91.3% in the United States, 66–92.7% in Italy, 92% in Israel, 84.1% in Canada, 81.7% in Malaysia, 79.1% in mainland China, and 71% in Lebanon and Brazil [8,9,10,11,12,13,14,15,16,17,18,19,20]. The prevalence of taking up at least one dose of COVID-19 vaccination was high among older adults in most parts of the world, for example, 79.1% in the United States (April 2021) [21], 94% in the United Kingdom (May 2021) [22], 94% in Singapore (October 2021) [23], and 80% in mainland China (November 2021) [24]. In contrast, older adults in Hong Kong reported a very low COVID-19 vaccination uptake during the study period (70–79 years: 38.4%, ≥80 years: 13.38% in October 2021) [3], which is much lower than their younger counterparts (20–69 years: 57.84–80.4%) [3]. Vaccine hesitancy among older adults is a significant challenge of COVID-19 control in Hong Kong.

The Hong Kong government has been implementing mass media campaigns to promote COVID-19 vaccination and providing incentives to motivate people to get vaccinated. There are some health promotions targeting older adults, including online videos and pamphlets emphasizing the high risk of COVID-19 mortality, and the safety and efficacy of COVID-19 vaccination for older adults. Good health promotional materials should be easy to understand, able to address the target audience’s greatest concern, and helpful in their decision making. It is important to understand whether older adults are satisfied with the vaccination materials produced by the government, as there were news reports suggesting that some health promotional materials have failed to address their greatest concerns related to the COVID-19 vaccine [25]. 

As COVID-19 vaccination uptake plateaued in mid-2021, many countries began to experiment with incentives to motivate people to get vaccinated. The most promising incentive option was guaranteed cash payment. A trial in Sweden found that guaranteed cash payment increased COVID-19 vaccination uptake by 4% [26]. Some other countries provided non-cash rewards, such as free rice/eggs in mainland China, hummus in Israel, and blenders in India [27]. However, there was a dearth of studies evaluating the effectiveness of such incentives. Another common option is lotteries with cash/non-cash prizes. In the United States, Ohio offered a million-dollar lottery, but the evaluation showed no benefits towards increasing COVID-19 vaccination [28]. Hong Kong government also offered some incentives to motivate people to get vaccinated, such as lottery for winning prizes, exemption of regular COVID-19 testing, allowing visits to elderly homes and hospitals, entering bars/clubs, reducing quarantine duration, and walk-in vaccination services without prior booking [3]. One study showed that vaccine passports for cross-border travel was most valued by people aged 18 years or above in Hong Kong, followed by allowance to enter entertainment venues [29]. However, these incentives were not designed specifically for older adults in Hong Kong. It is necessary to understand the impact of these incentives on increasing COVID-19 vaccination among older adults. 

Facilitators and barriers to receive COVID-19 vaccination among older adults might be different from those of their younger counterparts. It is also important to understand determinants of COVID-19 vaccination specific to older adults to develop health promotional campaigns to cater to their needs. To our knowledge, at least 12 studies have investigated factors associated with willingness to take up COVID-19 vaccination among older adults [9,10,11,12,13,14,15,16,17,18,19,20], while fewer studies have looked at factors influencing actual uptake in this group [10,22]. Facilitators identified by these studies included a history of seasonal influenza vaccination [18,19,20]; perceived higher risk and severer consequences of COVID-19 [10,11,12,14,17,19,20]; belief in vaccine efficacy and other benefits [10,13,14,15,17]; trust in vaccination information obtained from social media, friends, or family members [11,14]; higher social support [11]; and trust in the healthcare system, healthcare workers, or the government [11,14]. Barriers to receive COVID-19 vaccination found by these studies included ethnicity (Hispanic, Black, or African American) [9,16], lower education or income [9,19,20], aged over 70 years [13,19], and concerns about safety and side-effects of the vaccines [10,15,16,19,20]. Most of these facilitators and barriers were similar to those in the general population. Recent data showed that 75% of older adults in Hong Kong are suffering from one or more chronic diseases [30]. It is possible that older adults would be concerned about the interactions between COVID-19 vaccination and older age/chronic diseases, such as worrying about older age or presence of chronic diseases possibly leading to poorer vaccine efficacy and more severe side effects. However, no study has investigated whether such concerns are barriers of COVID-19 vaccination uptake among older adults.

In addition, since there is more than one type of COVID-19 vaccine available in Hong Kong, people may experience “choice overload” and find it difficult to select one for themselves [31]. Decisional conflicts refer to personal uncertainty about which option to take when there are competing options. Decisional conflicts were barriers of health service utilization in previous studies, especially when there was more than one option available [32]. This study tested whether decisional conflicts in choosing a COVID-19 vaccine could be a barrier among older adults.

To address these knowledge gaps, this study investigated the impacts of incentives and health promotional materials provided by the government on the completion of the primary COVID-19 vaccination series among a random sample of community-dwelling adults aged 65 years or above in Hong Kong. This study also investigated the associations of perceptions related to COVID-19 vaccination and decisional conflict with COVID-19 vaccination uptake. We hypothesized that older adults who perceived higher impacts of governmental incentives, were more satisfied with health promotional materials produced by the government, had perceptions supporting COVID-19 vaccination, and had a lower level of decisional conflict regarding the choice of vaccines would be more likely to complete the primary vaccination series.

## 2. Materials and Methods

### 2.1. Study Design

**Figure 1:**
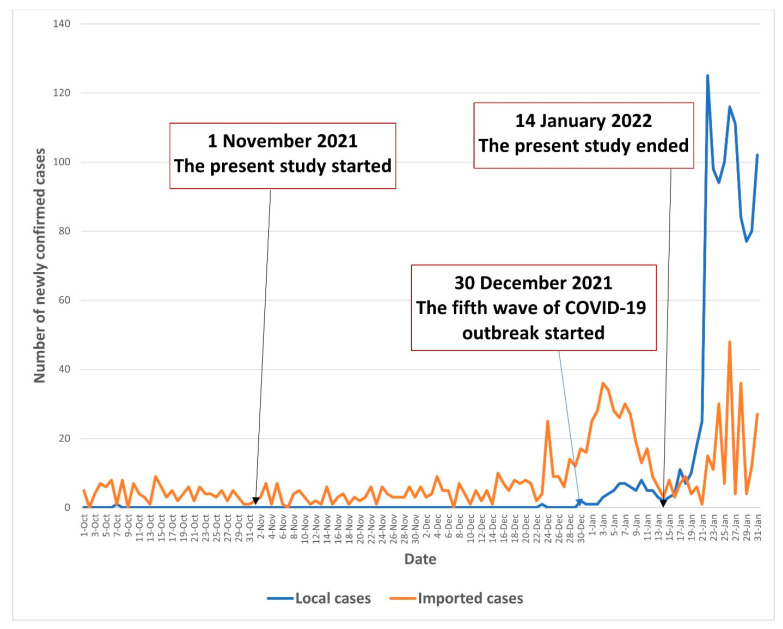
The COVID-19 situation in Hong Kong during the study period.

A random telephone survey among community-dwelling Chinese-speaking adults aged 65 years or above in Hong Kong was conducted between 1 November 2021 and 14 January 2022. The COVID-19 pandemic in Hong Kong was stable between 1 November and 29 December 2021; there were almost no local cases during this period. By the end of the recruitment period (30 December 2021 to 14 January 2022), Hong Kong entered the fifth wave of the COVID-19 outbreak, with the number of newly confirmed local cases starting to increase (1–8 during this period). The COVID-19 situation in Hong Kong during the study period is illustrated in Figure 1.

### 2.2. Participants and Sample Size Planning

Participants were community-dwelling Chinese-speaking individuals aged ≥65 years who had a Hong Kong ID card. Those who were not able to communicate effectively with the interviewers were excluded. Our target sample size was 400. We assumed the completion rate of primary COVID-19 vaccination series to be 50%. Given a statistical power of 0.80 and an alpha value of 0.05, and assuming the completion rate in the reference group (without a facilitating condition) to be 10–40%, the sample size could detect the smallest odds ratio of 1.76 between people with and without a facilitating condition (PASS 11.0, NCSS LLC). According to the Hong Kong census data in 2021, 18.2% of Hong Kong people were 65 years or above [33]. Assuming the participation rate of valid households to be 55–60%, we needed to screen about 4000 households in order to recruit 400 eligible participants. The same approach for sample size planning was used in published studies [34,35,36,37]. 

### 2.3. Data Collection

All household telephone numbers listed in the most up-to-date telephone directories (about 350,000) were input into an Excel file. Using the function of “select random cells”, a total of 4000 household telephone numbers were randomly selected. Trained interviewers conducted the telephone calls during 6–10 pm on weekdays and 2–9 pm on Saturdays to avoid under-sampling of working individuals. If no one in the household answered the initial call, four more follow-up calls were made on different days and hours before the household was considered to be non-valid (one without an eligible participant). If there was more than one person in the household who was aged ≥65 years, the one whose last birthday was closest to the interview date was invited to join the study. This was to avoid data contamination and introduction of extra confounding factors. Eligibility was screened. Prospective eligible participants were briefed about the study. Guarantees were made on anonymity, the right to quit at any time, and that refusal would have no consequences. Participants were asked the following: (1) whether they understand the briefing and (2) whether they are willing to participate. Since there was no face-to-face contact and the study was anonymous, verbal instead of written informed consent was sought. The interviewers signed a form pledging that the participants have been fully informed about the study. The same procedures have been used in previous random telephone surveys targeting local older adults [38,39]. The telephone interview took about 20 min to complete. A total of 3963 households were called; 698 had an eligible older adult, 258 prospective participants refused to participate in the study, and 440 completed the telephone survey. The response rate was 63% (440/698). No incentives were provided to participants. Ethics approval was obtained from the Survey and Behavioral Research Ethics Committee of the Chinese University of Hong Kong (SBRE-19-187).

### 2.4. Measures

#### 2.4.1. Development of the Questionnaire

With reference to a qualitative study exploring barriers to receive COVID-19 vaccination among 31 older adults (24 women and 7 men) aged 65 years or above in Hong Kong [40], a panel of researchers in public health, behavioral health, and vaccination behaviors was formed to design the questionnaire used in this study. The questionnaire was pilot tested among 10 older adults to assess clarity and readability. All the older adults participating in the pilot study indicated that the items of the questionnaire were easy to understand, and that the length of the questionnaire was acceptable. The Cronbach’s alpha of the Attitude Scale, the Subjective Norm Scale, and the Decisional Conflict Scale in the pilot testing were 0.79, 0.73, and 0.89, respectively. These older adults did not participate in the actual survey. The panel finalized the questionnaire based on their comments.

#### 2.4.2. Background Characteristics

Participants reported sociodemographic characteristics (age, gender, education level, relationship status, current employment status, monthly household income, and whether they were living alone) and lifestyles (smoking and binge drinking in the past year). Health conditions (presence of chronic conditions and history of COVID-19) and history of seasonal influenza and pneumococcal vaccination were also collected.

#### 2.4.3. COVID-19 Vaccination Uptake

Participants reported the number of doses and types of COVID-19 vaccines received, presence of side effects, and severity of such side effects. We defined completion of primary COVID-19 vaccination series as receiving two doses of inactivated or mRNA vaccines.

#### 2.4.4. Perceived Impacts of Incentives Provided by the Government on Increasing One’s Motivation to Receive COVID-19 Vaccination

Participants were asked about the level of impact of five incentives provided by the government on increasing one’s motivation to receive COVID-19 vaccination (response categories: 1 = almost none, 2 = small, 3 = moderate, and 4 = large). These incentives included the following: (1) lottery for winning prizes, (2) visiting mainland China or other places without quarantine, (3) allowing visits to elderly homes and hospitals without COVID-19 testing, (4) entering bars or clubs, and (5) walk-in vaccination services without prior booking. 

#### 2.4.5. Satisfaction with COVID-19 Vaccination Health Promotional Materials Produced by the Government

We adapted questions validated in the Chinese population to assess satisfaction of COVID-19 vaccination health promotional materials produced by the government [41]. Participants were asked whether the information presented by vaccination promotional materials produced by the government (e.g., advertisements, posters, and web pages) was easy to understand, and whether these materials could address their concerns related to COVID-19 vaccination and help them make a decision on whether to receive the vaccine (response categories: yes, no, and uncertain).

#### 2.4.6. Perceptions Related to COVID-19 Vaccination

A 4-item Attitude Scale and a 2-item Subjective Norm Scale were constructed for this study by summing up individual item scores (response categories: 1 = disagree, 2 = neutral, and 3 = agree). Higher scores on these scales indicated a more negative attitudes toward COVID-19 vaccination and perceptions that significant others were not supportive of their COVID-19 vaccination. The single item measuring perceived behavioral control was adapted from the measurement validated in the Chinese population [41,42]. Higher scores in these measurements indicated perceived higher behavioral control of taking up COVID-19 vaccination. The measurement of decisional conflict was adapted from the validated Chinese version of the SURE test version of the Decisional Conflict Scale [37]. Higher scores on the scale indicated less decisional conflict. The Cronbach’s alpha of the Attitude Scale, the Subjective Norm Scale, and the Decisional Conflict Scale were 0.84, 0.72, and 0.94, respectively.

### 2.5. Statistical Analysis

Frequency distribution of background characteristics, COVID-19 vaccination uptake, and other independent variables of interest are presented. Mean and standard deviation (SD) of item and scale scores representing perceived impacts of governmental incentives and perceptions related to COVID-19 vaccination are also presented. Cronbach’s alpha for the scales was obtained by using reliability tests. Principal component analysis with varimax rotation was used to perform exploratory factor analysis. We compared COVID-19 vaccination uptake, distribution of independent variables of interest between subgroups of participants aged 65–74 years and those aged ≥75 years, and between those who were recruited before (1 November to 30 December 2020) and after (1–14 January 2021) the fifth wave of the COVID-19 outbreak. Self-reported completion of primary COVID-19 vaccination series was the dependent variable. Univariate logistic regression models first assessed the significance between background characteristics and the dependent variable. Item and scale scores representing perceived impact of governmental incentives and perceptions related to COVID-19 vaccination were used as the independent variables. We fitted a single logistic regression model to obtain adjusted odds ratios (AOR), which involved one of these independent variables and all significant background characteristics in univariate analysis. In addition, a multivariate logistic regression model was fitted considering all variables with *p* < 0.05 as candidates. Sub-group analyses were performed among subgroups of participants aged 65–74 years and those aged ≥75 years. Cohen’s d was calculated to reflect the effect size of the associations between independent variables of interest and the dependent variable. There were no missing values for the participants who completed the survey. SPSS version 26.0 (IBM Corp) was used for data analysis, with *p* < 0.05 considered as statistically significant.

## 3. Results

### 3.1. Background Characteristics of the Participants

About half of the participants were 65–69 years old (49.8%) and had not received secondary education (42.5%). The majority of them were female (61.1%), married or cohabiting with a partner (74.3%), without full-time or part-time work (85.7%), and had a monthly income of less than HK$20,000 (US$2580) (74.5%). About 20% of them were living alone (18.4%). Regarding their health conditions, 60.9% of them reported having at least one chronic condition, and 1.8% had a history of COVID-19. At the time of the survey, 60% and 25.2% of participants had previously taken up seasonal influenza and pneumococcal vaccination. (Table 1)

### 3.2. COVID-19 Vaccination Uptake

Among the participants, 58.4% had completed the primary COVID-19 vaccination series, and 2.3% had received one dose of the COVID-19 vaccine (Table 2). Among vaccinated participants (n = 267), 142 (53.2%) had received CoronaVac (Sinovac, Beijing, China), 124 (46.4%) had received Comirnaty (Fosun-BioNTech, Jiangsu, China and Germany), and one participant could not remember which type of vaccine he had received. The side-effects of COVID-19 vaccination reported by the participants were mild (not at all: n = 118, very mild: n = 106, mild: n = 30, moderate: n = 7, severe: n = 6). Compared to participants recruited before the fifth wave of the COVID-19 outbreak (1 November to 30 December 2021), those recruited after the outbreak reported higher completion of primary COVID-19 vaccination series (80.6% versus 56.7%, *p* = 0.03) (Supplementary S1). 

### 3.3. Descriptive Statistics of Independent Variables of Interest 

The majority of the participants reported that governmental incentives had almost no impact on increasing their motivation to receive COVID-19 vaccination, such as lottery for winning prizes (86.8%), visiting mainland China or other places without quarantine (56.4%), allowing visits to elderly homes and hospitals without COVID-19 testing (70.0%), entering bars or clubs (89.8%), or walk-in vaccination services without prior booking (76.4%). Although 88.9% of participants believed that the information presented by vaccination promotional materials produced by the government were easy to understand, less than half thought that such materials were able to address their concerns related to COVID-19 vaccination (41.6%), or help them make a decision on whether to receive the vaccine (48.2%). Item responses and item and scale scores of perceptions related to COVID-19 vaccination are shown in Table 2. Compared to those aged 65–74 years, participants aged ≥75 years exhibited a more negative attitudes toward COVID-19 vaccination. Fewer participants aged ≥75 years believed that the information presented by health promotional materials was easy to understand compared to their younger counterparts. Both age groups perceived similar impacts of governmental incentives on increasing their motivations to receive COVID-19 vaccination (Supplementary S2). Compared to participants recruited before the fifth wave of the COVID-19 outbreak (1 November to 30 December 2021), those recruited after the outbreak perceived a lower impact of some governmental incentives, including lottery for winning prizes, visiting mainland China or other places without quarantine, and walk-in vaccination services for older adults without prior booking (Supplementary S1). 

### 3.4. Factors Associated with Completion of the Primary COVID-19 Vaccination Series

In univariate analysis, older age and receiving Comprehensive Social Security Assistance (CSSA) were associated with lower completion of the primary COVID-19 vaccination series. Higher education level, with full-time/part-time work, and history of pneumococcal vaccination were positively associated with the dependent variable (Table 3).

After adjustment for significant background characteristics, perceived higher impacts of governmental incentives on increasing their motivation to get vaccinated was associated with higher completion of the primary COVID-19 vaccination series. These incentives included lottery for winning prizes (AOR: 1.41, 95% CI: 1.05–1.90), visiting mainland China or other places without quarantine (AOR: 1.43, 95% CI: 1.20–1.71), allowing visits to elderly homes and hospitals without COVID-19 testing (AOR: 1.78, 95% CI: 1.40–2.27), entering bars or clubs (AOR: 2.02, 95% CI: 1.23–3.33), or walk-in vaccination services without prior booking (AOR: 1.31, 95% CI: 1.06–1.61). Belief that vaccination materials produced by the government could address their concerns related to COVID-19 vaccination (AOR: 4.21, 95% CI: 2.70–6.58) and were helpful for them in making a decision on whether to receive vaccination (AOR: 4.74, 95% CI: 3.07–7.34) were also positively associated with the dependent variable. In addition, more negative attitudes (AOR: 0.62, 95% CI: 0.55–0.69), and perceptions that significant others would not support them to receive COVID-19 vaccination (AOR: 0.55, 95% CI: 0.46–0.67) were associated with lower completion of the primary COVID-19 vaccination series, while perceived higher behavioral control (AOR: 6.77, 95% CI: 2.29–20.04) and less decisional conflict (AOR: 2.53, 95% CI: 2.02–3.17) when choosing a vaccine were positively associated with the dependent variable (Table 4). In the summary logistic regression model considering all variables with *p* < 0.05 in univariate analysis as candidates, current employment status; receiving comprehensive social security assistance; history of pneumococcal vaccination; perceived impact of allowing mainland China or other places without quarantine; and attitudes, perceived subjective norm, perceived behavioral control, and decisional conflict related to COVID-19 vaccination remained statistically significant (Table 5). 

Subgroup analysis showed that perceived higher impacts of governmental incentives were associated with a higher completion rate of primary COVID-19 vaccination series among participants aged 65–74 years. However, such associations were non-significant among participants aged ≥75 years. The associations of satisfaction with health promotional materials and perceptions related to COVID-19 vaccination with completion of primary vaccination series were similar between the two subgroups of participants (Supplementary S2). 

## 4. Discussion

This is one of the first studies to look at determinants of COVID-19 vaccination uptake among community-dwelling older adults in Hong Kong. Among the participants, only 58.4% had completed the primary COVID-19 vaccination series. The completion rate was especially low among people aged 75 years or above (43.2%). The completion rate observed by this study was consistent with the official data reported by the Hong Kong government during the study period, and was much lower than that of the United States, the United Kingdom, Singapore, and mainland China [21,22,23,24]. At that time, Hong Kong was severely hit by the fifth wave of the COVID-19 outbreak, mainly caused by Omicron sub-variant BA.2. As of 15 March 2022, over 95% of the 4066 cumulative COVID-19 deaths were aged 60 years or above [43]. Most of the COVID-19 deaths occurred among older adults who had not completed the primary vaccination series [43]. Hence, there is an urgent need to increase COVID-19 vaccination uptake among older adults in Hong Kong. 

Compared to adults aged 65–69 and 70–74 years, those aged 75 years or above reported significantly lower COVID-19 vaccination uptake. A similar trend was observed in Malaysia and Canada [13,19]. Therefore, future health promotional campaigns should target older age groups. In line with the findings in previous studies [9,19,20], lower socio-economic status (e.g., lower education, receiving CSSA) was associated with lower COVID-19 vaccination uptake. Health communication messages for older adults should be straightforward and easy to understand for people with a low level of literacy. Having a full-time or part-time work was associated with higher COVID-19 vaccination uptake. One possible reason is that some local employers require their employees to either receive COVID-19 vaccination or take up regular COVID-19 testing [44]. History of pneumococcal vaccination was also associated with higher COVID-19 vaccination uptake, which was similar to findings among older adults in Italy, Canada, and Saudi Araba [18,19,20]. Older adults with experience of receiving pneumococcal vaccination might have a stronger motivation towards using vaccines to prevent emerging infectious diseases. 

This study has numerous practical implications towards strengthening the ongoing COVID-19 vaccination promotional campaigns in Hong Kong. Previous studies suggested that incentives/rewards for vaccination would be more effective when people’s receipt was certain, the incentives were delivered immediately, and people valued such incentives [45]. Although guaranteed cash payment and non-cash rewards have been effective in increasing COVID-19 vaccination uptake in some countries (e.g., Sweden) [26], they have not been implemented in Hong Kong. Incentives offered by the Hong Kong government have not been guaranteed or delivered immediately, which might limit their effectiveness. Among the incentives, visiting mainland China or other places was most valued by older adults. Such findings were similar to those of people aged 18 years or above in Hong Kong [29]. Many local older adults migrated to Hong Kong from mainland China when they were young, and had close connections with family members living in mainland China. Therefore, this incentive was appealing to them. However, such an incentive could be controversial. One study conducted among older adults in Italy during the same period found a drop in vaccination acceptance after the mandatory introduction of “green pass” (COVID-19 immunization certificate to access public spaces) [8]. The implementation of such an incentive must be accompanied by effective education and information strategies towards the target population [8]. Very few participants perceived a lottery for winning prizes to have any impact on increasing their motivation to receive COVID-19 vaccination. In the United States, Ohio offered a million dollar lottery, but the evaluation did not find any benefits towards increasing COVID-19 vaccination [28]. It is likely that people prefer guarantees as incentives for vaccination rather than gambles [27]. Allowance to enter bars/clubs was considered to be a useful incentive for COVID-19 vaccination among people aged 18 years or above in Hong Kong [29]. However, very few older adults found such an incentive appealing, as they might not visit such entertainment venues as often as their younger counterparts might. In addition, sub-group analysis showed that none of these incentives worked well among participants aged ≥15 years, as their associations with COVID-19 vaccination completion were non-significant. Therefore, it is important for the government to put forward incentives tailored to the needs of older adults, especially for those aged 75 years or above. It is recommended that the government consult and obtain feedback from older adults when developing incentives to motivate COVID-19 vaccination uptake.

Less than half of the participants believed that the existing health promotional materials could address their greatest concern related to COVID-19 vaccination or that they were helpful for them in making decisions. It is necessary to improve the contents of such health promotional materials, as higher satisfaction with these materials was associated with higher COVID-19 vaccination uptake among older adults. Our results also suggested ways to improve these materials. Health promotional materials should address older adults’ concerns regarding the interactions between older age, chronic conditions, and COVID-19 vaccination, as such concerns were negatively associated with COVID-19 vaccination uptake. Recent evidence showed that the efficacy in preventing hospital admission and the prevalence of side effects of CoronaVac and Comirnaty were similar when comparing younger and older people [46,47,48]. Moreover, a study among Hong Kong people showed that presence of a chronic condition would not lead to more side effects of COVID-19 vaccination [49]. There was no evidence showing that COVID-19 vaccination would negatively affect the control of chronic diseases. This evidence should be included in health promotional materials produced by the government in order to address older adults’ concerns. Currently, most public health interventions are developed using a top-down approach where end users’ involvement is limited, with most intervention components designed and directed by academics and healthcare professionals [50]. These interventions are standardized without considering the needs of end users from their perspectives [51]. Making use of co-creation, which refers to collaborative public health intervention development by academics alongside end users [52,53,54], may be helpful in improving these materials. Such an approach is considered to be a promising and more efficient solution for addressing complex issues and fostering behavioral change [52,55]. In addition, family doctors and family members should be involved in vaccination promotion, as their support was found to be important for COVID-19 vaccination uptake among older adults. The phenomenon of “choice overload” might exist among older adults, as about 30% were not sure about which type of COVID-19 vaccine was suitable for them. Less decisional conflict was associated with higher COVID-19 vaccination uptake. Efficacies and side effects of different COVID-19 vaccines available in Hong Kong can be compared in a table, which may make it easier for older adults to compare features across products [31]. 

The study was based on a random and population-based sample, which was a strength of this study. However, it has some limitations. First, some measurement tools (e.g., attitudes toward COVID-19 vaccination) were constructed for our study, as there were no validated tools for older adults in Hong Kong. This was one major limitation of our study. The reliability of these measurements was acceptable both in pilot and the actual study. Second, the gender distribution among our participants was similar to the census data of people aged ≥65 years in Hong Kong [33]. However, people who were ≥75 years were under-sampled in this study. Third, policies related to COVID-19 vaccination are being updated rapidly in response to the quickly changing pandemic. Such changes have impacts on COVID-19 vaccination uptake among older adults in Hong Kong. Our findings are most applicable to the situation when COVID-19 was stable and under control in Hong Kong. Fourth, this study did not include older residents of residential care homes. The finding may not be generalizable to the entire population of older adults in Hong Kong. COVID-19 vaccination for these older residents was arranged by residential care homes. Different determinants might apply to this group of older adults. Fifth, selection bias existed due to non-response. We were not able to collect information from those who refused to participate in the study. Our response rate was comparable to other random telephone surveys on vaccination behaviors among older adults [38,39]. Furthermore, data were self-reported and verification was not feasible. Recall bias existed. Moreover, causality could not be established as this was a cross-sectional study.

## 5. Conclusions

Community-dwelling older adults aged 65 years or above in Hong Kong reported a low prevalence of completing the primary COVID-19 vaccination series. The completion rate was even lower among those aged 75 years or above. Existing governmental incentives had a limited impact on increasing older adults’ COVID-19 vaccination uptake. Health promotional materials produced by the government might not adequately address older adults’ greatest concerns and facilitate their decision making related to COVID-19 vaccination uptake. Considering interactions between COVID-19 vaccination and older age/chronic diseases and making use of a co-creation approach might be useful in improving these health promotional materials.

## Figures and Tables

**Table 1:** Background characteristics of the participants (n = 440).

	N	%
**Sociodemographic characteristics**		
Age group, years		
65–69	219	49.8
70–74	147	33.4
≥75	74	16.8
Gender		
Male	171	38.9
Female	269	61.1
Relationship status		
Currently single	113	25.7
Married or cohabiting with a partner	327	74.3
Education level		
Primary or below	187	42.5
Secondary	209	47.5
Tertiary or above	44	10.0
Current employment status		
Unemployed/retired/housewife	377	85.7
Full-time/part-time	63	14.3
Monthly household income, HK$ (US$)		
<20,000 (2580)	328	74.5
≥20,000 (2580)	58	13.2
Refused to disclose	54	12.3
Receiving Comprehensive Social Security Assistance (CSSA) ^1^		
No	408	92.7
Yes	32	7.3
Living alone		
No	359	81.6
Yes	81	18.4
**Lifestyles and health conditions**		
Smoking in the past year		
No	409	93.0
Yes	31	7.0
Binge drinking in the past year		
No	430	97.7
Yes	10	2.3
Presence of the following chronic conditions, yes		
Hypertension	212	48.2
Chronic cardiovascular diseases	46	10.5
Chronic lung diseases	8	1.8
Chronic liver diseases	10	2.3
Chronic kidney diseases	3	0.7
Diabetes Mellitus	83	18.9
Any of above	268	60.9
History of COVID-19		
No	432	98.2
Yes	8	1.8
**Uptake of other vaccination**		
History of seasonal influenza vaccination		
No	176	40.0
Yes	264	60.0
History of pneumococcal vaccination		
No	329	74.8
Yes	111	25.2

^1^ CSSA: a governmental financial support scheme providing a safety net for those who cannot support themselves financially.

**Table 2:** Descriptive statistics of COVID-19 vaccination uptake and independent variables of interest (n = 440).

	N	%
**COVID-19 vaccination uptake**		
Number of doses of COVID-19 vaccination received by the participants		
0	173	39.3
1	10	2.3
2	257	58.4
**Perceived impacts of incentives provided by the government on increasing one’s motivation to receive COVID-19 vaccination**		
Lottery for winning prizes		
Almost none	382	86.8
Small	18	4.1
Moderate	22	5.0
Large	18	4.1
Item score, mean (SD)	1.3	0.7
Visiting mainland China or other places without quarantine		
Almost none	248	56.4
Small	50	11.4
Moderate	59	13.4
Large	83	18.9
Item score, mean (SD)	2.0	1.2
Allowing visits to elderly homes and hospitals without COVID-19 testing		
Almost none	308	70.0
Small	45	10.2
Moderate	43	9.8
Large	44	10.0
Item score, mean (SD)	1.6	1.0
Entering bars or clubs		
Almost none	395	89.8
Small	25	5.7
Moderate	17	3.9
Large	3	0.7
Item score, mean (SD)	1.2	0.5
Walk-in vaccination services for older adults without prior booking		
Almost none	336	76.4
Small	21	4.8
Moderate	34	7.7
Large	49	11.1
Item score, mean (SD)	1.5	1.0
**Satisfaction with COVID-19 vaccination health promotional materials (e.g., advertisements, posters, and others) produced by the government**		
Whether the information is easy to understand		
No/uncertain	49	11.1
Yes	391	88.9
Whether the materials can address your concerns related to COVID-19 vaccination		
No/uncertain	257	58.4
Yes	183	41.6
Whether the materials are helpful for you in making a decision on whether to receive a COVID-19 vaccine		
No/uncertain	228	51.8
Yes	212	48.2
**Perceptions related to COVID-19 vaccination**		
Attitudes toward COVID-19 vaccination, agree		
The protection offered by the COVID-19 vaccination is weaker among people with older age	75	17.0
The side effects of COVID-19 vaccination are more severe among people with older age	125	28.4
Presence of chronic diseases could decrease the protection of COVID-19 vaccination	135	30.7
COVID-19 vaccination could negatively affect the control of existing chronic conditions	119	27.0
Attitude Scale ^1^, mean (SD)	7.6	2.5
Subjective norm related to COVID-19 vaccination, agree		
Your family doctors would not support you to take up COVID-19 vaccination	34	7.7
Your children or other family members would not support you to take up COVID-19 vaccination	86	19.5
Subjective Norm Scale ^2^, mean (SD)	3.4	1.1
Perceived behavioral control to take up COVID-19 vaccination agree		
You are confident to receive COVID-19 vaccination if you want to	414	94.1
Item score, mean (SD)	2.9	0.4
Decisional conflicts, agree		
You are sure about which type of COVID-19 vaccine is suitable for you	302	68.6
You are sure about which type of COVID-19 vaccine you should choose	307	69.8
Decisional Conflict Scale ^3^, mean (SD)	5.3	1.1

^1^ Attitude Scale: 4 items, Cronbach’s alpha: 0.84; one factor was identified by exploratory factor analysis, accounting for 56.1% of total variance. ^2^ Subjective Norm Scale: 2 items, Cronbach’s alpha: 0.72; one factor was identified by exploratory factor analysis, accounting for 67.8% of total variance. ^3^ Decisional Conflict Scale: 2 items, Cronbach’s alpha: 0.94; one factor was identified by exploratory factor analysis, accounting for 94.6% of total variance.

**Table 3:** Associations between background characteristics and completion of primary COVID-19 vaccination series among older adults in Hong Kong (n = 440).

	Completion of Primary COVID-19 Vaccination Series (%)	OR (95% CI)	*p*-Value	Cohen’s d
**Sociodemographic characteristics**				
Age group, years				
65–69	59.4	1.0		
70–74	64.6	1.25 (0.81–1.93)	0.31	0.12
≥75	43.2	0.52 (0.31–0.89)	0.02	−0.36
Gender				
Male	57.9	1.0		
Female	58.7	1.04 (0.70–1.53)	0.86	0.02
Relationship status				
Currently single	54.9	1.0		
Married or cohabiting with a partner	59.6	1.22 (0.79–1.87)	0.38	0.11
Education level				
Primary or below	54.5	1.0		
Secondary	58.9	1.19 (0.80–1.78)	0.39	0.10
Tertiary or above	72.7	2.22 (1.08–4.58)	0.03	0.44
Current employment status				
Unemployed/retired/housewife	56.0	1.0		
Full-time/part-time	73.0	2.13 (1.18–3.85)	0.01	0.42
Monthly household income, HK$ (US$)				
<20,000 (2580)	57.0	1.0		
≥20,000 (2580)	58.6	1.07 (0.61–1.88)	0.82	0.04
Refused to disclose	66.7	1.51 (0.82–2.77)	0.18	0.23
Receiving Comprehensive Social Security Assistance (CSSA) ^1^				
No	60.3	1.0		
Yes	34.4	0.35 (0.16–0.74)	0.01	−0.58
Living alone				
No	60.2	1.0		
Yes	50.6	0.68 (0.42–1.10)	0.12	−0.21
**Lifestyles and health conditions**				
Smoking in the past year				
No	59.2	1.0		
Yes	48.4	0.65 (0.31–1.34)	0.24	−0.24
Binge drinking in the past year				
No	58.4	1.0		
Yes	60.0	1.07 (0.30–3.85)	0.92	0.04
Presence of chronic conditions				
No	63.4	1.0		
Yes	55.2	0.71 (0.48–1.06)	0.09	−0.19
History of COVID-19				
No	58.8	1.0		
Yes	37.5	0.42 (0.10–1.78)	0.24	−0.48
**Uptake of other vaccination**				
History of seasonal influenza vaccination				
No	53.4	1.0		
Yes	61.7	1.41 (0.96–2.07)	0.08	0.19
History of pneumococcal vaccination				
No	54.5	1.0		
Yes	70.3	1.98 (1.25–3.14)	0.004	0.38

^1^ CSSA: a governmental financial support scheme providing a safety net for those who cannot support hemselves financially. OR—crude odds ratios; CI—confidence interval.

**Table 4:** Factors associated with completion of primary COVID-19 vaccination series among older adults in Hong Kong (n = 440).

	OR (95% CI)	*p* Values	Cohen’s d	AOR (95% CI)	*p*-Value	Cohen’s d
**Perceived impacts of incentives provided by the government on increasing one’s motivation to receive COVID-19 vaccination**						
Lottery for winning prizes	1.36 (1.02–1.81)	0.04	0.17	1.41 (1.05–1.90)	0.02	0.19
Visiting mainland China or other places without quarantine	1.42 (1.20–1.68)	<0.001	0.19	1.43 (1.20–1.71)	<0.001	0.20
Allowing visits to elderly homes and hospitals without COVID-19 testing	1.69 (1.34–2.11)	<0.001	0.29	1.78 (1.40–2.27)	<0.001	0.32
Entering bars or clubs	1.81 (1.13–2.90)	0.01	0.33	2.02 (1.23–3.33)	0.01	0.39
Walk-in vaccination services for older adults without prior booking	1.27 (1.05–1.55)	0.02	0.13	1.31 (1.06–1.61)	0.01	0.15
**Satisfaction with COVID-19 vaccination health promotional materials (e.g., advertisements, posters, and others) produced by the government**						
Whether the information is easy to understand						
No/uncertain	1.0			1.0		
Yes	1.69 (0.93–3.06)	0.09	0.29	1.48 (0.77–2.85)	0.24	0.22
Whether the materials can address your concerns related to COVID-19 vaccination						
No/uncertain	1.0			1.0		
Yes	4.08 (2.67–6.23)	<0.001	0.78	4.21 (2.70–6.58)	<0.001	0.79
Whether the materials are helpful for you in making a decision on whether to receive a COVID-19 vaccine						
No/uncertain	1.0			1.0		
Yes	4.54 (3.00–6.85)	<0.001	0.83	4.74 (3.07–7.34)	<0.001	0.86
**Perceptions related to COVID-19 vaccination**						
Attitude Scale	0.62 (0.56–0.68)	<0.001	−0.26	0.62 (0.55–0.69)	<0.001	−0.26
Subjective Norm Scale	0.56 (0.47–0.68)	<0.001	−0.32	0.55 (0.46–0.67)	<0.001	−0.32
Perceived behavioral control to take up COVID-19 vaccination	7.49 (2.51–22.34)	<0.001	1.11	6.77 (2.29–20.04)	0.001	1.05
Decisional Conflict Scale	2.50 (2.01–3.11)	<0.001	0.51	2.53 (2.02–3.17)	<0.001	0.51

OR—crude odds ratios; CI—confidence interval; AOR—adjusted odds ratios; odds ratios adjusted for significant background characteristics are listed in Table 3.

**Table 5:** Summary model of factors associated with completion of primary COVID-19 vaccination series among older adults in Hong Kong (n = 440).

	AOR (95% CI)	*p*-Value	Cohen’s d
Age group, years			
65–69	1.0		
70–74	2.01 (1.06–3.83)	0.03	0.39
≥75	0.75 (0.35–1.58)	0.45	−0.16
Education level			
Primary or below	1.0		
Secondary	0.92 (0.53–1.59)	0.76	−0.05
Tertiary or above	1.67 (0.58–4.82)	0.34	0.28
Current employment status			
Unemployed/retired/housewife	1.0		
Full-time/part-time	3.01 (1.28–7.10)	0.01	0.61
Receiving Comprehensive Social Security Assistance (CSSA)			
No	1.0		
Yes	0.30 (0.10–0.95)	0.01	−0.66
History of pneumococcal vaccination			
No	1.0		
Yes	2.07 (1.06–4.02)	0.03	0.40
**Perceived impacts of incentives provided by the government on increasing one’s motivation to receive COVID-19 vaccination**			
Lottery for winning prizes	1.01 (0.66–1.26)	0.95	0.01
Visiting mainland China or other places without quarantine	1.96 (1.30–2.95)	0.01	0.37
Allowing visits to elderly homes and hospitals without COVID-19 testing	0.93 (0.69–1.26)	0.64	−0.04
Entering bars or clubs	1.17 (0.61–2.25)	0.64	0.09
Walk-in vaccination services for older adults without prior booking	1.03 (0.75–1.41)	0.86	0.02
**Satisfaction with COVID-19 vaccination health promotional materials (e.g., advertisements, posters, and others) produced by the government**			
Whether the materials can address your concerns related to COVID-19 vaccination			
No/uncertain	1.0		
Yes	1.06 (0.44–2.52)	0.90	0.03
Whether the materials are helpful for you in making a decision on whether to receive a COVID-19 vaccine			
No/uncertain	1.0		
Yes	1.83 (0.79–4.25)	0.16	0.33
**Perceptions related to COVID-19 vaccination**			
Attitude Scale	0.68 (0.60–0.77)	<0.001	−0.21
Subjective Norm Scale	0.71 (0.55–0.90)	0.01	−0.19
Perceived behavioral control to take up COVID-19 vaccination	5.07 (1.50–17.16)	0.01	0.90
Decisional Conflict Scale	1.98 (1.53–2.57)	<0.001	0.38

AOR—adjusted odds ratios; odds ratios were obtained by fitting a multivariate logistic regression model considering all variables with *p* < 0.05 in univariate analysis.

## Data Availability

The data presented in this study are available from the corresponding author upon request. The data are not publicly available as they contain sensitive personal information.

## Supplementary Materials

The following supporting information can be downloaded at: https://www.mdpi.com/article/10.3390/vaccines10050732/s1, Supplementary S1: Comparing COVID-19 vaccination uptake before and after the 5th wave; Supplementary S2: Comparing participants aged 65–74 years with those aged 75 years or above.
