# Supplementary material for: Governmental Incentives, Satisfaction with Health Promotional Materials, and COVID-19 Vaccination Uptake among Community-Dwelling Older Adults in Hong Kong: A Random Telephone Survey
Source: Vaccines (Basel). 2022 May 6;10(5):732. doi: 10.3390/vaccines10050732 (PMC9147504; doi:10.3390/vaccines10050732)
Supplement: Supplementary file 1 [file vaccines-10-00732-s001.zip › Supplementary 1 Comparing COVID-19 vaccination uptake before and after the 5th wave.pdf]

Supplementary S1. Comparing COVID-19 vaccination uptake and independent variables of interest before and after the fifth wave of COVID-19 outbreak in Hong Kong.

Table S1. Comparing COVID-19 vaccination uptake and other independent variables of interest between participants recruited before and after the fifth wave of COVID-19 outbreak in Hong Kong.

| | Before the 5th wave of outbreak (n=409) | After the 5th wave of outbreak (n=31) | P values |
|---|---|---|---|
| | n (%) | n (%) | |
| **COVID-19 vaccination uptake** | | | |
| Number of doses of COVID-19 vaccination received by the participants | | | |
| 0 | 167 (40.8) | 6 (19.4) | |
| 1 | 10 (2.4) | 0 (0.0) | |
| 2 | 232 (56.7) | 25 (80.6) | 0.03 |
| **Perceived impacts of incentives provided by the government in increasing one's motivation to receive COVID-19 vaccination** | | | |
| Lottery for winning prizes | | | |
| Almost none | 352 (92.1) | 30 (96.8) | |
| Small | 18 (4.4) | 0 (0.0) | |
| Moderate | 21 (5.1) | 1 (3.2) | |
| Large | 18 (4.4) | 0 (0.0) | 0.35 |
| Item score, mean (SD) | 1.3 (0.7) | 1.1 (0.4) | 0.01 |
| Visiting mainland China or other places without quarantine | | | |
| Almost none | 229 (56.0) | 19 (61.3) | |
| Small | 42 (10.3) | 8 (25.8) | |
| Moderate | 56 (13.7) | 3 (9.7) | |
| Large | 82 (20.0) | 1 (3.2) | 0.01 |
| Item score, mean (SD) | 2.0 (1.3) | 1.6 (0.8) | 0.01 |
| Allowing visit of elderly homes and hospitals without COVID-19 testing | | | |
| Almost none | 288 (70.4) | 20 (64.5) | |
| Small | 39 (9.5) | 6 (19.4) | |
| Moderate | 39 (9.5) | 4 (12.9) | |
| Large | 43 (10.5) | 1 (3.2) | 0.19 |
| Item score, mean (SD) | 1.6 (1.0) | 1.6 (0.9) | 0.78 |
| Entering bars or clubs | | | |
| Almost none | 367 (89.7) | 28 (90.3) | |
| Small | 24 (5.9) | 1 (3.2) | |
| Moderate | 15 (3.7) | 2 (6.5) | |
| Large | 3 (0.7) | 0 (0.0) | 0.76 |

| | | | |
|---|---|---|---|
| Item score, mean (SD) | 1.2 (0.5) | 1.2 (0.5) | 0.94 |
| Walk-in vaccination services for older adults without prior booking | | | |
| Almost none | 307 (75.1) | 29 (93.5) | |
| Small | 20 (4.9) | 1 (3.2) | |
| Moderate | 34 (8.3) | 0 (0.0) | |
| Large | 48 (11.7) | 1 (3.2) | 0.12 |
| Item score, mean (SD) | 1.7 (1.1) | 1.1 (0.6) | <0.001 |
| **Satisfaction of COVID-19 vaccination health promotion materials (e.g., advertisement, poster, and others) produced by the government** | | | |
| Whether the information are easy to understand | | | |
| No/uncertain | 46 (11.2) | 3 (9.7) | |
| Yes | 363 (88.8) | 28 (90.3) | 0.79 |
| Whether the materials can address your concerns related to COVID-19 vaccination | | | |
| No/uncertain | 243 (59.4) | 14 (45.2) | |
| Yes | 166 (40.6) | 17 (54.8) | 0.12 |
| Whether the materials are helpful for you to make decision to receive a COVID-19 vaccine | | | |
| No/uncertain | 214 (52.3) | 14 (45.2) | |
| Yes | 195 (47.7) | 17 (54.8) | 0.44 |
| **Perceptions related to COVID-19 vaccination** | | | |
| Attitudes toward COVID-19 vaccination, agree | | | |
| The protection offered by the COVID-19 vaccination is weaker among people with older age | 70 (17.1) | 5 (16.1) | 0.89 |
| The level of side effects of COVID-19 vaccination is severer among people with older age | 122 (29.8) | 3 (9.7) | 0.02 |
| Presence of chronic diseases would decrease the protection of COVID-19 vaccination | 128 (31.3) | 7 (22.6) | 0.31 |
| COVID-19 vaccination would negatively affect the control of existing chronic conditions | 114 (27.9) | 5 (16.1) | 0.16 |
| Attitudes Scale [1], mean (SD) | 7.7 (2.5) | 6.9 (2.4) | 0.09 |
| Subjective norm related to COVID-19 vaccination, agree | | | |
| Your family doctors would not support you to take up COVID-19 vaccination | 31 (7.6) | 3 (9.7) | 0.67 |

| | | | |
|---|---|---|---|
| Your children or other family members would not support you to take up COVID-19 vaccination | 78 (19.1) | 8 (25.8) | 0.36 |
| Subjective Norm Scale [2], mean (SD) | 3.4 (1.1) | 3.2 (1.3) | 0.34 |
| | | | |
| Perceived behavioral control to take up COVID-19 vaccination, agree | | | |
| You are confident to receive COVID-19 vaccination if you want to | 384 (92.8) | 30 (96.8) | 0.51 |
| Item score, mean (SD) | 2.9 (0.4) | 3.0 (0.2) | 0.39 |
| | | | |
| Decisional conflicts, agree | | | |
| You are sure about which type of COVID-19 vaccine is suitable for you | 277 (67.7) | 25 (80.6) | 0.14 |
| You are sure about which type of COVID-19 vaccine you should choose | 282 (68.9) | 25 (80.6) | 0.17 |
| Decisional Conflict Scale [3], mean (SD) | 5.3 (1.1) | 5.5 (1.0) | 0.17 |

P values were obtained by using Chi-square tests (for categorical variables) and independent sample t-tests (for continuous variables)

[1] Attitudes Scale: 4 items, Cronbach's alpha: 0.84, one factor was identified by exploratory factor analysis, explaining for 56.1% of total variance

[2] Subjective Norm Scale: 2 items, Cronbach's alpha: 0.72, one factor was identified by exploratory factor analysis, explaining for 67.8% of total variance

[3] Decisional Conflict Scale, 2 items, Cronbach's alpha: 0.94, one factor was identified by exploratory factor analysis, explaining for 94.6% of total variance
