# Supplementary material for: Governmental Incentives, Satisfaction with Health Promotional Materials, and COVID-19 Vaccination Uptake among Community-Dwelling Older Adults in Hong Kong: A Random Telephone Survey
Source: Vaccines (Basel). 2022 May 6;10(5):732. doi: 10.3390/vaccines10050732 (PMC9147504; doi:10.3390/vaccines10050732)
Supplement: Supplementary file 1 [file vaccines-10-00732-s001.zip › Supplementary 2 Comparing participants aged 65-74 years with those aged 75 years or above.pdf]

Supplementary S2. Comparing participants aged 65-74 years with those aged 75 years or above.

Table S2. Comparing background characteristics of the participants aged 65-74 years with those aged 75 years or above.

	Participants aged 65-74 years (n=366)	Participants aged 75 years or above (n=74)	P values
	n (%)	n (%)	
**Sociodemographic characteristics**			
Gender			
Male	138 (37.7)	33 (44.6)	
Female	228 (62.3)	41 (55.4)	0.27
Relationship status			
Currently single	84 (23.0)	29 (39.2)	
Married or cohabited with a partner	282 (77.0)	45 (60.8)	0.004
Education level			
Primary or below	155 (42.3)	32 (43.2)	
Secondary	175 (47.8)	34 (45.9)	
Tertiary or above	36 (9.8)	8 (10.8)	0.94
Current employment status			
Unemployed/retired/housewife	305 (83.3)	72 (97.3)	
Full-time/part-time	61 (16.7)	2 (2.7)	0.002
Monthly household income, HK$ (US$)			
<20,000 (2,580)	272 (74.3)	56 (75.7)	
≥20,000 (2,580)	49 (13.4)	9 (12.2)	
Refuse to disclose	45 (12.3)	9 (12.2)	0.96
Receiving Comprehensive Social Security Assistance (CSSA) [1]			
No	338 (92.3)	70 (94.6)	
Yes	28 (7.7)	4 (5.4)	0.50
Living alone			
No	309 (84.4)	50 (67.6)	
Yes	57 (15.6)	24 (32.4)	0.001
**Lifestyles and health conditions**			
Smoking in the past year			
No	342 (93.4)	67 (90.5)	
Yes	24 (6.6)	7 (9.5)	0.37
Binge drinking in the past year			
No	359 (98.1)	71 (95.9)	
Yes	7 (1.9)	3 (4.1)	0.26
Presence of the following chronic conditions, Yes			
Hypertension	172 (47.0)	40 (54.1)	0.27
Chronic cardiovascular diseases	35 (9.6)	11 (14.9)	0.17
Chronic lung diseases	6 (1.6)	2 (2.7)	0.53

Chronic liver diseases	10 (2.7)	0 (0.0)	0.15
Chronic kidney diseases	3 (0.8)	0 (0.0)	0.44
Diabetes Mellitus	67 (18.3)	16 (21.6)	0.51
Any of above	217 (59.3)	51 (68.9)	0.12
History of COVID-19			
No	361 (98.6)	71 (95.9)	
Yes	5 (1.4)	3 (4.1)	0.11
**Uptake of other vaccination**			
History of seasonal influenza vaccination			
No	155 (42.3)	21 (28.4)	
Yes	211 (57.7)	53 (71.6)	0.03
History of pneumococcal vaccination			
No	281 (76.8)	48 (64.9)	
Yes	85 (23.2)	26 (35.1)	0.03

1 CSSA: a governmental financial support scheme providing a safety net for those who cannot support themselves financially.

P values were obtained by using Chi-square tests (for categorical variables) and independent sample t-tests (for continuous variables)

Table S3. Comparing COVID-19 vaccination uptake and other independent variables of interest between participants aged 65-74 years and those aged 75 years or above.

	Participants aged 65-74 years (n=366)	Participants aged 75 years or above (n=74)	P values
	n (%)	n (%)	
**COVID-19 vaccination uptake**			
Number of doses of COVID-19 vaccination received by the participants			
0	134 (36.7)	38 (51.3)	
1	6 (1.6)	4 (5.4)	
2	225 (61.6)	32 (43.2)	0.01
**Perceived impacts of incentives provided by the government in increasing one's motivation to receive COVID-19 vaccination**			
Lottery for winning prizes			
Almost none	314 (85.8)	68 (91.9)	
Small	15 (4.1)	3 (4.1)	
Moderate	21 (5.7)	1 (1.4)	
Large	16 (4.4)	2 (2.7)	0.38
Item score, mean (SD)	1.3 (0.7)	1.2 (0.6)	0.08
Visiting mainland China or other places without quarantine			
Almost none	201 (54.9)	47 (63.5)	
Small	41 (11.2)	9 (12.2)	
Moderate	52 (14.2)	7 (9.5)	
Large	72 (19.7)	11 (14.9)	0.45
Item score, mean (SD)	2.0 (1.2)	1.8 (1.1)	0.14
Allowing visit of elderly homes and hospitals without COVID-19 testing			
Almost none	258 (70.5)	50 (67.6)	
Small	35 (9.6)	10 (13.5)	
Moderate	34 (9.3)	9 (12.2)	
Large	39 (10.7)	5 (6.8)	0.48
Item score, mean (SD)	1.6 (1.0)	1.6 (0.9)	0.88
Entering bars or clubs			
Almost none	329 (89.9)	66 (89.2)	
Small	21 (5.7)	4 (5.4)	
Moderate	13 (3.6)	4 (5.4)	
Large	3 (0.8)	0 (0.0)	0.76
Item score, mean (SD)	1.2 (0.5)	1.2 (0.5)	0.89
Walk-in vaccination services for older adults			

without prior booking			
Almost none	284 (77.6)	52 (70.3)	
Small	12 (3.3)	9 (12.2)	
Moderate	28 (7.7)	6 (8.1)	
Large	42 (11.5)	7 (9.5)	0.01
Item score, mean (SD)	1.5 (1.0)	1.6 (1.0)	0.78
**Satisfaction of COVID-19 vaccination health promotion materials (e.g., advertisement, poster, and others) produced by the government**			
Whether the information are easy to understand			
No/uncertain	34 (9.3)	15 (20.3)	
Yes	332 (90.7)	59 (79.7)	0.01
Whether the materials can address your concerns related to COVID-19 vaccination			
No/uncertain	211 (57.7)	46 (62.2)	
Yes	155 (42.3)	28 (37.8)	0.47
Whether the materials are helpful for you to make decision to receive a COVID-19 vaccine			
No/uncertain	188 (51.4)	40 (54.1)	
Yes	178 (48.6)	34 (45.9)	0.67
**Perceptions related to COVID-19 vaccination**			
Attitudes toward COVID-19 vaccination, agree			
The protection offered by the COVID-19 vaccination is weaker among people with older age	55 (15.0)	20 (27.0)	0.01
The level of side effects of COVID-19 vaccination is severer among people with older age	97 (26.5)	28 (37.8)	0.049
Presence of chronic diseases would decrease the protection of COVID-19 vaccination	107 (29.2)	28 (37.8)	0.14
COVID-19 vaccination would negatively affect the control of existing chronic conditions	92 (25.1)	27 (36.5)	0.045
Attitudes Scale [1], mean (SD)	7.5 (2.5)	8.4 (2.5)	0.004
Subjective norm related to COVID-19 vaccination, agree			
Your family doctors would not support you to take up COVID-19 vaccination	26 (7.1)	8 (10.8)	0.28
Your children or other family members would not support you to take up COVID-19 vaccination	68 (18.6)	18 (24.3)	0.26

Subjective Norm Scale [2], mean (SD)	3.3 (1.1)	3.5 (1.2)	0.19
Perceived behavioral control to take up COVID-19 vaccination, agree			
You are confident to receive COVID-19 vaccination if you want to	346 (94.5)	68 (91.9)	0.38
Item score, mean (SD)	2.9 (0.4)	2.9 (0.4)	0.62
Decisional conflicts, agree			
You are sure about which type of COVID-19 vaccine is suitable for you	259 (70.8)	43 (58.1)	0.03
You are sure about which type of COVID-19 vaccine you should choose	262 (71.6)	45 (60.8)	0.07
Decisional Conflict Scale [3], mean (SD)	5.3 (1.1)	5.1 (1.1)	0.10

[1] Attitudes Scale: 4 items, Cronbach's alpha: 0.84, one factor was identified by exploratory factor analysis, explaining for 56.1% of total variance

[2] Subjective Norm Scale: 2 items, Cronbach's alpha: 0.72, one factor was identified by exploratory factor analysis, explaining for 67.8% of total variance

[3] Decisional Conflict Scale, 2 items, Cronbach's alpha: 0.94, one factor was identified by exploratory factor analysis, explaining for 94.6% of total variance

P values were obtained by using Chi-square tests (for categorical variables) and independent sample t-tests (for continuous variables)

Table S4. Associations between background characteristics and completion of primary COVID-19 vaccination series among participants aged 65-74 years and 75 years or above.

	Participants aged 65-74 years		Participants aged 75 years or above	
	OR (95%CI)	P values	OR (95%CI)	P values
**Sociodemographic characteristics**				
Gender				
Male	1.0		1.0	
Female	0.90 (0.58, 1.39)	0.63	1.67 (0.65, 4.25)	0.29
Relationship status				
Currently single	1.0		1.0	
Married or cohabited with a partner	1.19 (0.72, 1.95)	0.50	0.89 (0.35, 2.31)	0.83
Education level				
Primary or below	1.0		1.0	
Secondary	1.26 (0.81, 1.96)	0.30	0.90 (0.34, 2.36)	0.82
Tertiary or above	3.91 (1.54, 9.93)	0.004	0.38 (0.07, 2.16)	0.27
Current employment status				
Unemployed/retired/ housewife	1.0		1.0	
Full-time/part-time	1.95 (1.06, 3.61)	0.03	1.32 (0.08, 21.99)	0.85
Monthly household income, HK$ (US$)				
<20,000 (2,580)	1.0		1.0	
≥20,000 (2,580)	1.02 (0.55, 1.91)	0.94	1.24 (0.30, 5.12)	0.77
Refuse to disclose	1.30 (0.67, 2.52)	0.44	3.09 (0.70, 13.66)	0.14
Receiving Comprehensive Social Security Assistance (CSSA) [1]				
No	1.0		1.0	
Yes	0.38 (0.17, 0.83)	0.02	N.A.	N.A.
Living alone				
No	1.0		1.0	
Yes	0.77 (0.43, 1.36)	0.37	0.70 (0.26, 1.91)	0.49
**Lifestyles and health conditions**				
Smoking in the past year				
No	1.0		1.0	
Yes	0.51 (0.22, 1.16)	0.11	1.86 (0.39, 8.96)	0.44
Binge drinking in the past year				
No	1.0		1.0	
Yes	0.46 (0.10, 2.10)	0.32	N.A.	N.A.

Presence of chronic conditions				
No	1.0		1.0	
Yes	0.63 (0.41, 0.98)	0.04	1.67 (0.60, 4.62)	0.33
History of COVID-19				
No	1.0		1.0	
Yes	0.41 (0.07, 2.50)	0.34	0.65 (0.06, 7.45)	0.73
**Uptake of other vaccination**				
History of seasonal influenza vaccination				
No	1.0		1.0	
Yes	1.48 (0.97, 2.26)	0.07	1.79 (0.62, 5.13)	0.28
History of pneumococcal vaccination				
No	1.0		1.0	
Yes	2.10 (1.23, 3.61)	0.01	2.49 (1.00, 6.62)	0.048

OR: crude odds ratios, CI: confidence interval

Table S5. Factors associated with completion of primary COVID-19 vaccination series among participants aged 65-74 years and 75 years or above.

	Participants aged 65-69 years				Participants aged 70 years or above			
	OR (95%CI)	P values	AOR (95%CI)	P values	OR (95%CI)	P values	AOR (95%CI)	P values
**Perceived impacts of incentives provided by the government in increasing one's motivation to receive COVID-19 vaccination**								
Lottery for winning prizes	1.38 (1.01, 1.89)	0.046	1.40 (1.01, 1.94)	0.04	1.04 (0.46, 2.36)	0.92	1.13 (0.49, 2.58)	0.78
Visiting mainland China or other places without quarantine	1.48 (1.23, 1.80)	<0.001	1.45 (1.18, 1.77)	<0.001	1.08 (0.72, 1.62)	0.71	1.08 (0.71, 1.64)	0.72
Allowing visit of elderly homes and hospitals without COVID-19 testing	1.74 (1.35, 2.24)	<0.001	1.76 (1.35, 2.30)	<0.001	1.59 (0.96, 2.64)	0.07	1.49 (0.88, 2.52)	0.14
Entering bars or clubs	1.97 (1.12, 3.49)	0.02	1.97 (1.11, 3.49)	0.02	1.50 (0.59, 3.86)	0.40	1.83 (0.69 ,4.82)	0.22
Walk-in vaccination services for older adults without prior booking	1.23 (1.00, 1.53)	0.06	1.24 (0.99, 1.55)	0.07	1.57 (0.96, 2.56)	0.07	1.44 (0.87, 2.38)	0.16
**Satisfaction of COVID-19 vaccination health promotion materials (e.g., advertisement, poster, and**								

**others) produced by the government**								
Whether the information are easy to understand								
No/uncertain	1.0		1.0		1.0		10	
Yes	1.47 (0.72, 2.99)	0.29	1.34 (0.62, 2.88)	0.46	1.69 (0.51, 5.54)	0.39	2.03 (0.58, 7.03)	0.27
Whether the materials can address your concerns related to COVID-19 vaccination								
No/uncertain	1.00		1.0		1.0		1.0	
Yes	3.88 (2.42, 6.20)	<0.001	4.20 (2.56, 6.90)	<0.001	5.36 (1.93, 14.87)	0.001	5.45 (1.91, 15.55)	0.002
Whether the materials are helpful for you to make decision on whether to receive a COVID-19 vaccine								
No/uncertain	1.0		1.0		1.0		1.0	
Yes	4.23 (2.68, 6.67)	<0.001	4.53 (2.79, 7.35)	<0.001	7.20 (2.56, 20.23)	<0.001	7.05 (2.36, 20.22)	<0.001
**Perceptions related to COVID-19 vaccination**								
Attitudes Scale	0.59 (0.53, 0.67)	<0.001	0.59 (0.53, 0.57)	<0.001	0.75 (0.60, 0.92)	0.01	0.77 (0.62, 0.96)	0.02
Subjective Norm Scale	0.55 (0.45, 0.67)	<0.001	0.53 (0.43, 0.66)	<0.001	0.65 (0.43, 0.99)	0.045	0.64 (0.42, 0.98)	0.04
Perceived behavioral control to take up COVID-19 vaccination	8.98 (2.38, 33.97)	0.001	8.12 (2.22, 29.69)	0.002	3.61 (0.52, 24.94)	0.19	3.17 (0.55, 25.16)	0.18
Decisional Conflict Scale	2.55 (2.00, 3.25)	<0.001	2.52 (1.96, 3.23)	<0.001	2.13 (1.28, 3.53)	0.004	2.14 (1.27, 3.58)	0.004

OR: crude odds ratios, CI: confidence interval

AOR: adjusted odds ratios, odds ratios adjusted for significant background characteristics listed in Table 3.
